# Advances in CAR T-cell therapy in bile duct, pancreatic, and gastric cancers

**DOI:** 10.3389/fimmu.2022.1025608

**Published:** 2022-10-06

**Authors:** Qiang Feng, Baozhen Sun, Tianyi Xue, Rong Li, Chao Lin, Yongjian Gao, Liqun Sun, Yue Zhuo, Dongxu Wang

**Affiliations:** ^1^ Department of Hepatobiliary and Pancreas Surgery, China - Japan Union Hospital of Jilin University, Changchun, China; ^2^ Laboratory Animal Center, College of Animal Science, Jilin University, Changchun, China; ^3^ School of Acupuncture-Moxi bustion and Tuina, Changchun University of Chinese Medicine, Changchun, China; ^4^ School of grain science and technology, Jilin Business and Technology College, Changchun, China; ^5^ Department of Gastrointestinal Colorectal and Anal Surgery, China-Japan Union Hospital of Jilin University, Changchun, China; ^6^ Department of Pathogenobiology, Jilin University Mycology Research Center, College of Basic Medical Sciences, Jilin University, Changchun, China

**Keywords:** CAR T cells, chimeric antigen receptors, immunotherapy, digestive tumors, xenograft models

## Abstract

Bile duct, pancreatic, and gastric cancers are deadly digestive system tumors with high malignancy and poor patient prognosis. The efficiencies of conventional surgical treatment, radiation therapy, and chemotherapy are limited. In contrast, chimeric antigen receptor (CAR) T-cell therapy represents a landmark therapeutic approach to antitumor immunity with great efficacy in treating several hematological malignancies. CAR T-cell therapy involves genetically engineering the expression of specific antibodies based on the patient’s T-cell surface and amplifying these antibodies to identify and target tumor-associated antigens. CAR T-cell therapy can effectively inhibit disease progression and improve the survival of patients with bile duct, pancreatic, and gastric cancers. The effectiveness of CAR T cells in tumor therapy can be validated using xenograft models, providing a scientific testing platform. In this study, we have reviewed the progress in CAR T-cell production and its development, focusing on the current status and optimization strategies for engineered CAR T cells in the bile duct, pancreatic, and gastric cancers.

## Introduction

Tumor treatment has long been an important topic of continuous research in medical science. Conventional surgery, chemotherapy, and radiation therapy can cure only a small number of early-stage tumors with low malignancy and impair the proliferation and differentiation of both normal cells and tumor cells (1). Bile duct, pancreatic, and gastric cancers are the most common digestive system tumors with high malignancy and poor patient outcomes from conventional treatment (2–4). Bile duct cancer (CCA) is a type of hepatobiliary cancer with a high mortality rate. Early-stage bile duct cancer can be removed surgically, whereas advanced cases can only be managed by bile duct drainage surgery (5). CCA tumors contain large numbers of a cluster of differentiation (CD)8+ T cells and programmed cell death-ligand 1 (PD-L1). Current immunotherapy strategies using immune checkpoint inhibitors (ICIs) have not shown satisfactory results in CCA (6). Pancreatic cancer is a common malignant tumor of the gastrointestinal (GI) tract. It has a worse prognosis than almost all other tumor types because of its low early diagnosis rate and high surgical mortality (3). Although patients with pancreatic cancer contain high PD-L1 levels, their immunogenicity is inherently poor, and do not respond well to systemic therapy consisting of vaccines and ICIs (7). Gastric cancer is the third most common cause of cancer death worldwide and shows high molecular and phenotypical heterogeneity (4). Gastric cancer cells contain a high content of CD8+ T cells. The results of treating patients with gastric cancer using immune checkpoint blockade vary greatly depending on a tumor microenvironment (TME) (8). Patients with gastric cancer suffer from poor prognoses with strong recurrence risks (9). Therefore, developing therapies targeting malignant tumors has become a crucial yet challenging area of oncology research.

Immunotherapy is an emerging tool used in cancer treatment (1). Chimeric antigen receptor (CAR) T-cell therapies have reached milestones in preclinical research and clinical treatment and are the most promising cancer immunotherapies available today (10). CAR T-cell therapy has shown promising efficacy for treating hematologic malignancies (11). Four commercially available CAR T-cell products, Kymriah, Yescarta, Tecartus, and Breyanzi, have been authorized by the U.S. Food and Drug Administration and the European Commission for patients who suffer from recurrent or refractory B-cell precursor asthmatic lymphoblastic leukemia and intractable large B-cell lymphoma (11–18). Currently, CAR T-cell therapy does not work well in solid tumors, and many characteristics of solid tumors pose great challenges for using CAR T-cell therapy. As these cancers are largely refractory to conventional treatment, bile duct, pancreatic, and gastric cancers have been treated with CAR T-cell therapy (19). In most preclinical studies, researchers have used xenograft models to test CAR T-cell therapy in treating bile duct, pancreatic, and gastric cancers (20).

In this study, we have highlighted CAR T-cell therapy in the bile duct, pancreatic, and gastric cancers, summarized existing studies, briefly discussed the role of xenograft models in CAR T research, and discussed the future of engineered CAR T cells for treating digestive system tumors.

## CAR T-cell therapy

CAR T-cell therapy is a perinatal T-cell therapy using a patient’s immune system for treatment, as T cells collected from a patient are designed genetically to express specific antigen-binding domains that bind with intracellular signaling domains on the surface of T cells to identify specific tumor antigens and amplify these T cells (21). Lymphatic clearance is performed to enable effective cell transplantation. CAR T-cell therapy is then reinfused into the patient so that the engineered T cells target the patient’s specific antigens (22). CAR T-cell therapy has breathed new life into the field of cancer immunotherapy.

### Engineered CAR protein structures

**Figure 1:**
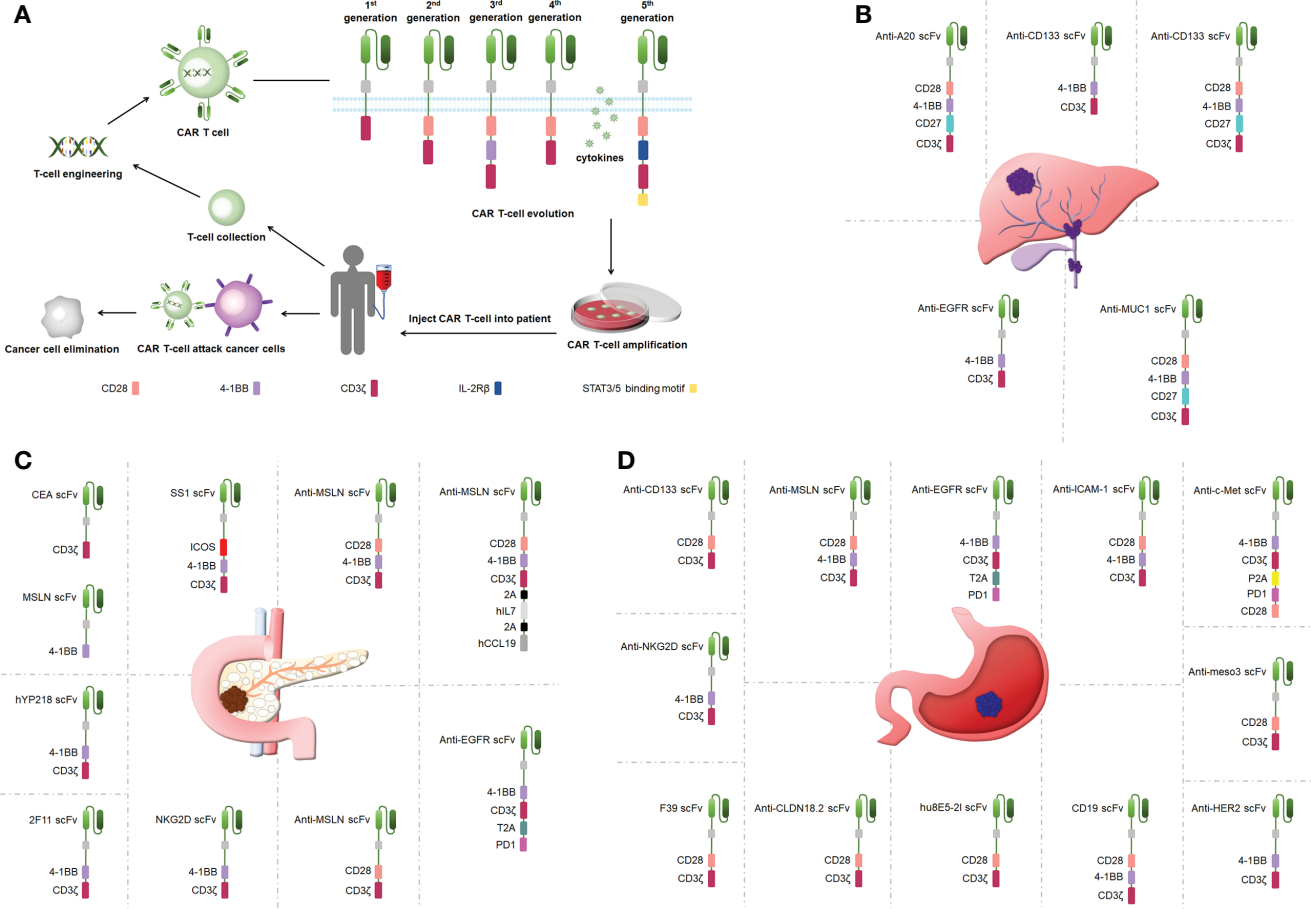
**(A)** CAR T-cell therapy and the structure of CARs. **(B)** Engineered CAR T cells in bile duct cancer. **(C)** Engineered CAR T cells in pancreatic cancer. **(D)** Engineered CAR T cells in gastric ancer.

A CAR is a synthetic receptor that activates T cells to target and recognizes tumor-associated antigens (TAAs), and a CAR binds to target antigens with no restrictions within the major histocompatibility complex (MHC) (19). A CAR works as a recombination receptor and includes one domain for extracellular antigen recognition, one transmembrane domain, and one intracellular signaling domain (**Figure 1A**) (23). The antigen-binding domain is typically found in the variable region of immunoglobulins (Igs) and consists of VH and VL chains linked by junctions into a single chain variability domain (scFv) (24). The “spacer domain” is usually an IgG1 hinge-CH_2_-CH_3_ Fc region with a constant structure between the scFv and spacer domain, which provides the flexibility to overcome spatial blockage (25). Most structural regions across the membrane consist of natural proteins, including CD3ζ, CD4, CD8α, and CD28 (26). The expression of CARs allows T cells to identify diverse cell surface antigens, expanding the range of tumor antigen targets (27). The structural properties of CARs increase the scope that CAR T-cell therapy can offer, providing more possibilities for tumor immunotherapy.

### The evolution of CAR engineering

CAR T cells have undergone five generations of updates and optimization thus far. The first-generation CARs provided signals *via* only one intracellular signaling domain, CD3ζ or FcRγ, and could not induce a considerable expansion of T cells (28). CD28 or 41BB was added between the scFv and CD3ζ chain in the second-generation CARs to strengthen the antitumor activity of CAR T cells (29). Third-generation CARs included more co-stimulatory structural domains including CD28 and 41BB as well as OX40 and CD40, in addition to exhibiting a stronger ability to activate and induce T-cell proliferation (30–32). The fourth-generation CARs added genes encoding cytokines (interleukin [IL]-12 and IL-15) to be released by the CARs to improve CAR T-cell survival in a TME (32). The fifth-generation CARs build on the second-generation CARs by adding cytoplasmic structural domains from the IL-2 receptor beta chain and signal transducers and activators of transcription (STAT)3/5 binding pattern, triggering three signals including T-cell receptors (CD3ζ structural domain), co-stimulatory factors (CD28 structural domain), and cytokines (Janus Kinase-STAT3/5 signaling) to improve the proliferation, survival, and antitumor activity of CAR T cells markedly (33). Overall, CAR T-cell therapy is upgraded mainly by optimizing the engineering of CARs, and the improvement in CAR performance effectively improves the success rate in treating tumors using CAR T cells.

### Mechanism of CAR-T therapy

T cells kill tumor cells by two mechanisms. One is the release of perforins and granzymes by cytokinesis. The other is tumor cell binding to tumor necrosis factors (TNFs) and thus undergoing apoptosis (34). When T cells are attached to an engineered CAR construct to form CAR T cells, they kill bile duct, pancreatic, and gastric cancer cells by three main mechanisms (**Figure S1**) (35, 36). These mechanisms are as follows: 1) CAR-T cells exert signal transduction and cell activation functions with the massive release of perforins and granzymes. Perforins specifically target tumor cell membranes in MHC- and Fas-independent manners to induce the formation of pores from which granzymes subsequently enter the interior of tumor cells. Granzymes trigger an enzyme chain reaction that leads to cell death by apoptosis. CAR T cells release more perforins/granzymes and have a higher affinity than natural T cells (37). 2) The main death receptor pathways are Fas and the corresponding death ligand FasL as well as TNFR and the corresponding death ligand TNF. The death receptor binds to specific death ligands, receives extracellular death signals, activates intracellular apoptotic mechanisms, and induces apoptosis. CAR-T cells are highly expressed on the surface of Fas or TNF ligands, which do not depend on antigen–antibody binding to induce apoptosis in a heterogeneous tumor environment (38). 3) CAR-T cells secret particular cytokines, such as interferon-gamma (IFN-γ). These cytokines can promote CAR-T activity, induce the tumor stroma expression of IFN- γ receptors, modify the cancer microenvironment, and enhance CAR T-cell activity against tumors, thereby mediating the killing of target cells (33). The complex and diverse mechanisms of CAR T-cell therapy potentially expand their application scope in immunotherapy oncology. Engineered CAR T cells may mediate multiple effector mechanisms simultaneously, enhancing their potential for tumor therapy.

## CAR T-cell therapy for digestive system tumors

CAR T-cell therapy is one of the most promising immunotherapeutic strategies (39). Most bile duct, pancreatic, and gastric cancer studies have applied only first-generation CAR T-cell therapies and have been limited by off-target toxicity (40). Current preclinical studies involving CAR T-cell therapies against these three GI tumors are focused on the refinement and optimization of CAR T-cell engineering.

### Bile duct cancer

CCA is treatment-resistant and prone to recurrence (41). One clinical study used a cocktail of CAR T therapies in patients with terminal CCA with the sequential infusion of epidermal growth factor receptor (EGFR)-specific and CD133-specific CAR T cells, which effectively inhibited tumor progression(**Figure 1B**) (42). Another study showed that CART–EGFR cell therapy was a secure and useful strategy for CCA (43). The targeted recognition and binding of tumor cell surface antigens is an important factor affecting the efficacy of CAR T cells in solid tumor therapies (44). Fourth-generation anti-CD133-CAR4 T cells tracking the antigen CD133 exhibited efficient antitumor effects. Fourth-generation A20-4G CAR T cells showed an antitumor activity by targeting integrin αvβ6, providing a viable experimental demonstration to support subsequent *in vivo* studies and clinical trials (45). Fourth-generation anti-mucin 1 (MUC1)-CAR4 T cells exhibited a specific killing activity by increasing the production of antitumor cytokines (46). This is the first study to show the therapeutic potential of anti-MUC1-CAR4 T cells for CCA. The antitumor activity that CAR T-cell therapy provides is better than traditional therapies, such as radiotherapy and chemotherapy, and can be applied to treat patients with CCA.

### Pancreatic cancer

Pancreatic cancer presents one of the toughest GI malignancies, and patients are usually diagnosed at a late stage because of a lack of obvious early symptoms; thus, novel therapeutic approaches need to be developed to improve the poor prognosis of patients with pancreatic cancer (47). CAR T-cell therapy efficacy may be limited by cell surface antigen-specific expression (48). Carcinoembryonic antigen (CEA) and mesothelin (MSLN) were highly expressed in pancreatic cancer, and dual-receptor CAR T cells targeting both antigens were found to target tumor sites precisely and reduce tumor load in pancreatic cancer mouse models (**Figure 1C**) (49). hYP218 CAR T cells directed to MSLN at proximal membrane surface sites induced durable antitumor immunity in mice (50). Notably, immunosuppressive cytokines could create barriers to tumor treatment (51). Inducible IL-18 CAR T cells were effective in inducing the regression of advanced pancreatic cancer in mouse models (52). In addition, IL-8 receptor-modified CARs with CD70 enhanced CAR T-cell efficacy in pancreatic cancer therapy (53). The combination production with IL-7 and chemokine (C-C pattern) ligand 19 (CCL19) 7×19 CAR T cells showed better antitumor activity against pancreatic cancer compared with regular CAR T-cell therapy (54). IL-7/CCL19 anti-MSLN CAR T cells eliminated malignant tumors *in situ* in mouse models (55). Targeting trophoblast surface antigen 2 (Trop2) using CAR T cells offered another potential resolution for pancreatic cancer (56). Similarly, CARs targeting chimeric programmed death receptor 1 increased survival in pancreatic cancer-bearing mice (57). Third-generation ICOSBBz CAR T cells inhibited pancreatic cancer progression while increasing CAR T-cell survival *in vivo (* 58). Microbial molecules can regulate immune cell motility, and valeric acid and butyric acid can increase the antitumor activity of CD8+ CAR T cells (59). Second-generation KD2-natural killer group 2D (NKG2D)-CAR T cells exhibited a stronger antitumor activity compared with first-generation NKG2D-CAR T cells in a pancreatic cancer xenograft model (20). Thus, CAR T-cell therapy can inhibit cancer progression in pancreatic cancer, circumvent drug resistance arising from conventional treatments, and has great potential in clinical applications in the future.

### Gastric cancer

Gastric cancer represents one of the most popular causes of death (60). As conventional surgical treatment and antitumor drugs have limited effects, CAR T-cell therapy can provide targeted immunotherapy to patients with gastric cancer without developing drug resistance and effectively control the progression and metastasis of gastric cancer (61). Targeting specific antigens on solid tumors can increase the efficacy of CAR T-cell therapy (39). Claudin18.2-specific CAR T cells can inhibit tumor growth with great efficacy and safety, and these studies were performed in mouse models (**Figure 1D**) (61, 62). Mesenchymal-epithelial transforming factor (c-Met) cMet-PD1/CD28 CAR showed no off-target toxicity in gastric cancer treatment (63). Targeting human epidermal growth factor receptor 2-expressing cells with CAR T cells suppressed tumors significantly (64). CAR T cells targeting MSLN region III (meso3 CAR, proximal membrane region) effectively mediated antitumor responses (65). Anti-MSLN CAR (M28z10) T cells also exhibited strong antitumor activity (66). Third-generation MSLN-CAR exhibited significant antitumor effects in gastric cancer patient-derived xenografts (PDXs) (67).

In addition to TAAs, the immunosuppression of cytokines in a TME represents an important candidate for optimizing CAR T-cell therapy. The TME affected a CAR T-cell activity in part *via* procedural cell death protein 1 (PD-1) (68). Third-generation bispecific Trop2/PD-L1 CAR T cells notably prevented the growth of tumors in mouse models (69). The CAR vector CARPD-L1z targeting PD-L1 inhibited tumor progression in a PDX model of gastric cancer (70). EGFR-CAR T cells, which secrete PD-1 scFv, could kill tumor cells in a gastric cancer model for a long time (71). Moreover, the scFv and an IL-2 fusion protein of scFv-IL2 CAR T cells enhanced antitumor activity against gastric cancer cells (72). CEA-CAR T cells showed strong antitumor activity in combination simultaneously with recombinant human IL-12, overcoming the limitations of each as an anticancer monomer (73). Similarly, the combination of cisplatin and anti-CD133 CAR T effectively inhibited the progression of gastric cancer (74). NKG2D-CAR T cell treatment combined with cisplatin also enhanced the antitumor activity against gastric cancer compared with cisplatin or NKG2D-CAR T cells alone (75). CAR T cells aimed at intercellular adhesion molecule 1 effectively inhibited disease progression (76). B7H3-specific CAR T cells with a humanized antigen recognition structural domain could effectively kill gastric cancer cells while targeting cancer stem cells to improve immune therapy efficacy, which was considered a potential strategy to treat gastric cancer (77). Optimized CAR T cells identify antigens specific to a patient’s particular gastric tumors, avoiding the killing of non-tumor cells caused by radiation and chemotherapy and providing more effective treatment for gastric cancer.

## Animal models for CAR T-cell therapies

The efficacy and safety of CAR T-cell therapy in cancer should be tested in experimental animal models. Animal models with xenotransplantation, *in situ* transplantation animal models, and gene editing animal models are the more commonly used models to study CAR T-cell therapy. In bile duct cancer, pancreatic cancer, and gastric cancer, xenograft animal models are commonly used to show the therapeutic efficacy of CAR T-cell therapy (20, 52, 59, 61, 70, 71).

**Figure 2:**
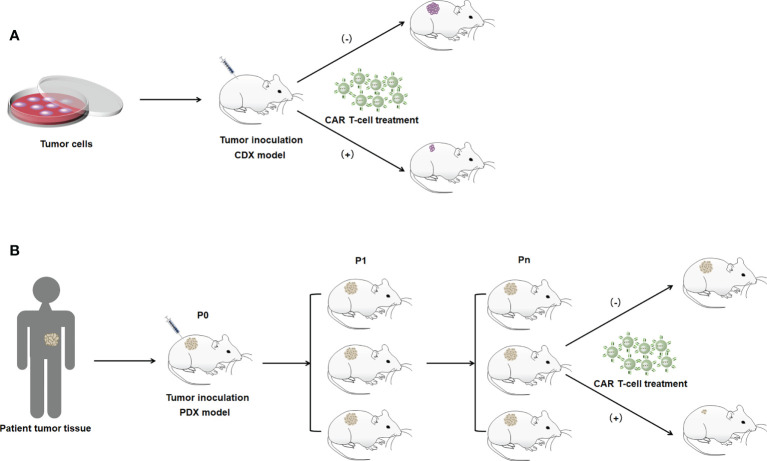
Illustration of the construction method of the xenograft model. **(A)** CDX model. **(B)** PDX model. P0 indicates mice directly inoculated with patient tumor tissue. P1 indicates mice with tumor tissues isolated from P0 generation mice for transplantation into the body. Pn indicates the Nth generation of mice passed on in accordance with the abovememtioned process.

Xenograft models are divided into cell-derived xenografts (CDXs) and PDXs based on the source of the xenograft (78). CDX models involve the transplantation of human-derived tumor cells into the subcutis of mice and other experimental animals after culturing cells *in vitro*, which allows the cells to be transplanted in large numbers under the same conditions (**Figure 2A**) (79). CDX models are widely used due to their short experimental cycles and high modeling success rate. However, PDX models have significant advantages over CDXs. PDXs tend to induce tumors more easily in animals. PDX models are generated by directly transplanting fresh human tumor tissue into immunodeficient mice. Because the tumor tissues are directly transplanted from patients into mice, genomic integrity and tumor heterogeneity are maximized (**Figure 2B**) (80). In addition, the tissues used for transplantation are not artificially cultured, and they simulate TME more accurately and have higher clinical similarity than CDX models, making PDXs the best animal model for tumors at this stage. However, they have to be implanted into immunodeficient mice. *In situ* transplantation is the inoculation of a human tumor into the host organ tissue corresponding to the primary site of the tumor (52). It has a microenvironment similar to human tumors, which is more suitable for tumor development and transfer of tumors. The *in situ* transplantation model better simulates the development and transfer process over clinical cancer and is an ideal model for tumor prevention and treatment and anti-metastasis research. Animal models are constructed by gene editing to mimic specific biological characteristics of human diseases to introduce target genes or delete and modify endogenous genes (81). Gene editing animals can accurately mimic human diseases caused by gene mutations, and contribute to the study of tumorigenesis mechanisms. In CAR T-cell therapy applications, constructing the appropriate animal models to build these methods that evaluate the therapeutic effects of engineered CAR T cells *in vivo* can be considered an important milestone to optimize CAR T-cell therapy that can be translated into clinical practice.

## Discussion

Bile duct cancer, pancreatic cancer, and gastric cancer are common digestive system tumors. Compared with other cancers, these digestive system tumors are prone to metastasis and have limited therapeutic effects from surgery, radiation, and chemotherapy (82). CAR T-cell therapy provides a more useful treatment for these types of cancers (42, 56, 67). CAR T-cell therapy acts as an innovative backbone for cancer therapy that expands the landscape of tumor immunotherapy (39). CAR T-cell therapy has promising clinical utility in hematologic diseases and has been approved as a commercial treatment (15, 17, 18). Nevertheless, CAR T-cell therapy is limited in solid tumors because of factors such as antigen escape, TME, and cell-related toxicity (83). CAR T cells can be programmed to improve antitumor efficacy in solid tumors while modulating the targeting and non-tumor toxicity of CAR T-cell therapy (84, 85). Because CAR T-cell therapy effectively inhibits the progression of tumors, overcomes patient drug resistance, improves poor prognosis, and prolongs patient survival in the bile duct, pancreatic, and gastric cancers, it is the best treatment for digestive system cancers until now (83, 86). In studies that optimize CAR T cells and use them to treat tumors, animal models are required to characterize and validate the anti-tumor effects of CAR T cells (67). Therefore, xenograft animal models, especially PDX models, should be selected to accurately mimic aspects of the human TME (80). The tumor tissue implanted in PDX models is directly derived from patients; therefore, it can maintain genomic integrity and tumor heterogeneity. PDX models can provide a stable scientific animal platform for CAR T-cell therapy research.

Presently, CAR T treatment strategies targeting solid tumors have been effectively improved. Notably, engineered CARs using gene editing technology have the potential of enhancing CAR T-cell therapy for the treatment of solid tumors (85). Gene editing technology and CAR T-cell therapy target tumor surface-specific antigens for precision therapy (87). Compared with traditional CAR T therapies, it significantly enhances the therapeutic effect on tumors while effectively avoiding the damage caused by off-target effects. Therefore, coupling gene editing technology and CAR T-cell therapy will increase the effectiveness of cancer treatments. Further optimization and modification are required to establish better immunotherapy strategies for CAR T-cell therapies which improve the therapeutic efficacy of CAR T-cell therapies for solid tumors by minimizing the issues of targeting and non-tumor toxicity to effectively suppress or eliminate tumors.

## Conclusion

In this review, we presented many designed and optimized CAR T-cell therapies in three digestive system cancers (bile duct cancer, pancreatic cancer, and gastric cancer). CAR T-cell therapy has been applied in these three digestive system tumors with promising results as a potential strategy for immunotherapy of solid tumors and has great oncological value. New strategies and treatment options for CAR T-cell therapy are under development. They can offer a promising road to safer and more successful immunotherapy of solid tumors.

## Author contributions

QF, BS, and DW wrote the manuscript. QF, BS, TX, RL,CL, YG, LS, YZ, and DW collected the references and prepared figures. All authors contributed to the article and approved the submitted version.

## Funding

This work was supported by the Natural Science Foundation of Jilin Province (20210101310JC), Central University Basic Scientific Research Fund (2019JCKT70), Scientific Research Project of Jilin University Key Laboratory ([2019]004), Project of Jilin Science and Technology Department 20220505033ZP, Jilin Science and Technology Development Program20220505033ZP, 202002006JC and 202101010JC.

## Conflict of interest

The authors declare that the research was conducted in the absence of any commercial or financial relationships that could be construed as a potential conflict of interest.

## Publisher’s note

All claims expressed in this article are solely those of the authors and do not necessarily represent those of their affiliated organizations, or those of the publisher, the editors and the reviewers. Any product that may be evaluated in this article, or claim that may be made by its manufacturer, is not guaranteed or endorsed by the publisher.

## Supplementary material

Supplementary Figure 1 Three main mechanisms of CAR T cell killing bile duct, pancreatic and gastric cancer cells.
