# Corrigendum: Advances in CAR T-cell therapy in bile duct, pancreatic, and gastric cancers

**DOI:** 10.3389/fimmu.2022.1082984

**Published:** 2022-12-20

**Authors:** Qiang Feng, Baozhen Sun, Tianyi Xue, Rong Li, Chao Lin, Yongjian Gao, Liqun Sun, Yue Zhuo, Dongxu Wang

**Affiliations:** ^1^ Department of Hepatobiliary and Pancreas Surgery, China - Japan Union Hospital of Jilin University, Changchun, China; ^2^ Laboratory Animal Center, College of Animal Science, Jilin University, Changchun, China; ^3^ School of Acupuncture-Moxi bustion and Tuina, Changchun University of Chinese Medicine, Changchun, China; ^4^ School of Grain Science and Technology, Jilin Business and Technology College, Changchun, China; ^5^ Department of Gastrointestinal Colorectal and Anal Surgery, China-Japan Union Hospital of Jilin University, Changchun, China; ^6^ Department of Pathogenobiology, Jilin University Mycology Research Center, College of Basic Medical Sciences, Jilin University, Changchun, China

**Keywords:** CAR T cells, chimeric antigen receptors, immunotherapy, digestive tumors, xenograft models

In the published article, the reference for Immunotherapy is an emerging tool used in cancer treatment (1) was incorrectly written as Ruhlmann CH, Iversen TZ, Okera M, Muhic A, Kristensen G, Feyer P, et al. Multinational study exploring patients’ perceptions of side-effects induced by chemo-radiotherapy. Radiother Oncol (2015) 117(2):333–7. doi: 10.1016/j.radonc.2015.09.014. It should be Mohanty R, Chowdhury CR, Arega S, Sen P, Ganguly P, Ganguly N. CAR T cell therapy: A new era for cancer treatment (Review). Oncol Rep (2019) 42(6):2183-95. Epub 2019/10/04. doi: 10.3892/or.2019.7335.

In the published article, the reference for The fourth-generation CARs added genes encoding cytokines [interleukin (IL)-12 and IL-15] to be released by the CARs to improve CAR T-cell survival in a TME (32) was incorrectly written as Haso W, Lee DW, Shah NN, Stetler-Stevenson M, Yuan CM, Pastan IH, et al. Anti-Cd22-chimeric antigen receptors targeting B-cell precursor acute lymphoblastic leukemia. Blood (2013) 121(7):1165–74. doi: 10.1182/blood-2012-06-438002. It should be Chmielewski M, Kopecky C, Hombach AA, Abken H. IL-12 release by engineered T cells expressing chimeric antigen receptors can effectively muster an antigen-independent macrophage response on tumor cells that have shut down tumor antigen expression. Cancer Res (2011) 71(17):5697-706. Epub 2011/07/12. doi: 10.1158/0008-5472.Can-11-0103.

In the published article, the reference for The fifth-generation CARs build on the second-generation CARs by adding cytoplasmic structural domains from the IL-2 receptor beta chain and signal transducers and activators of transcription (STAT)3/5 binding pattern, triggering three signals including T-cell receptors (CD3ζ structural domain), costimulatory factors (CD28 structural domain), and cytokines (Janus kinase-STAT3/5 signaling) to improve the proliferation, survival, and antitumor activity of CAR T cells markedly (33) was incorrectly written as Chmielewski M, Kopecky C, Hombach AA, Abken H. IL-12 release by engineered T cells expressing chimeric antigen receptors can effectively muster an antigen-independent macrophage response on tumor cells that have shut down tumor antigen expression. Cancer Res (2011) 71(17):5697–706. doi: 10.1158/0008-5472.Can-11-0103. It should be Kagoya Y, Tanaka S, Guo T, Anczurowski M, Wang CH, Saso K, et al. A novel chimeric antigen receptor containing a JAK-STAT signaling domain mediates superior antitumor effects. Nat Med (2018) 24(3):352-9. Epub 2018/02/06. doi: 10.1038/nm.4478.

In the published article, the reference for As conventional surgical treatment and antitumor drugs have limited effects, CAR T-cell therapy can provide targeted immunotherapy to patients with gastric cancer without developing drug resistance and effectively control the progression and metastasis of gastric cancer (62) was incorrectly written as Durães C, Almeida GM, Seruca R, Oliveira C, Carneiro F. Biomarkers for gastric cancer: Prognostic, predictive or targets of therapy? Virchows Arch (2014) 464(3):367–78. doi: 10.1007/s00428-013-1533-y. It should be Jiang H, Shi Z, Wang P, Wang C, Yang L, Du G, et al. Claudin18.2-specific chimeric antigen receptor engineered T cells for the treatment of gastric cancer. J Natl Cancer Inst (2019) 111(4):409-18. Epub 2018/09/12. doi: 10.1093/jnci/djy134.

In the published article, the reference for Animal models are constructed by gene editing to mimic specific biological characteristics of human diseases to introduce target genes or delete and modify endogenous genes (83) was incorrectly written as Hu W, Lazar MA. Modelling metabolic diseases and drug response using stem cells and organoids. Nat Rev Endocrinol (2022) 1–16. doi: 10.1038/s41574-022-00733-z. It should be Platt RJ, Chen S, Zhou Y, Yim MJ, Swiech L, Kempton HR, et al. CRISPR-Cas9 knockin mice for genome editing and cancer modeling. Cell (2014) 159(2):440-55. Epub 2014/09/30. doi: 10.1016/j.cell.2014.09.014.

The authors apologize for these errors and state that these do not change the scientific conclusions of the article in any way. The original article has been updated.

