# Supplementary figures and images for: Advances in CAR T-cell therapy in bile duct, pancreatic, and gastric cancers
Source: Front Immunol. 2022 Oct 6;13:1025608. doi: 10.3389/fimmu.2022.1025608 (PMC9628995; doi:10.3389/fimmu.2022.1025608)

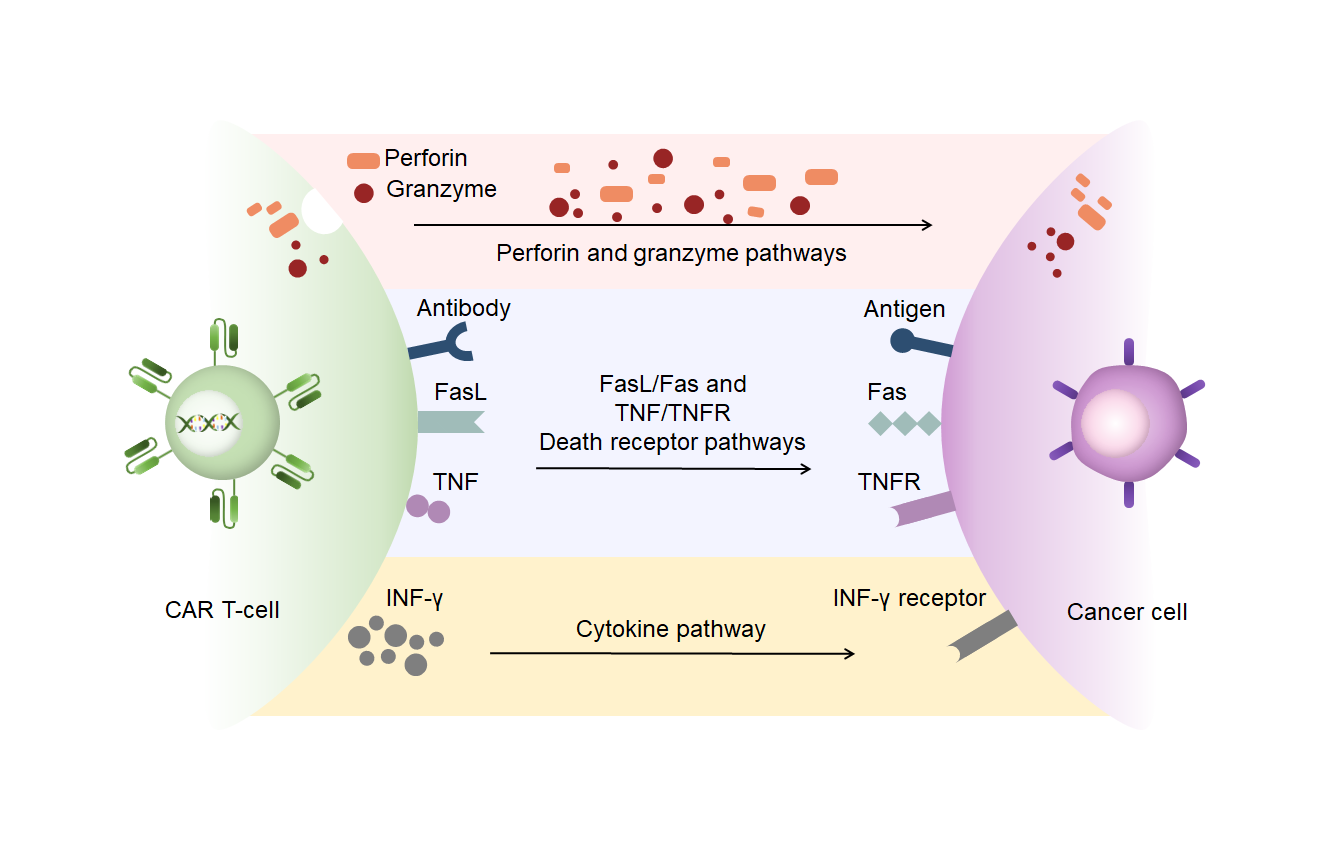

Supplement: Supplementary Figure 1 — Three main mechanisms of CAR T cell killing bile duct, pancreatic and gastric cancer cells. [file Image_1.tif]
