# Supplementary material for: Advances in CAR T-cell therapy in bile duct, pancreatic, and gastric cancers
Source: Front Immunol. 2022 Oct 6;13:1025608. doi: 10.3389/fimmu.2022.1025608 (PMC9628995; doi:10.3389/fimmu.2022.1025608)
Supplement: Supplementary file 2 [file Table_1.docx]

**Table S1: Clinical application of CAR T-cell therapy in bile duct cancer, pancreatic cancer and gastric cancer.**

| Cancer | Biological Interventions | Study Phase | Clinical Treatment Effect | First Posted Time | References /ClinicalTrials.gov Identifier |
| --- | --- | --- | --- | --- | --- |
| Bile duct cancer | CART-EGFR | Phase 1/Phase 2 | 1 patient achieved complete response and 10 patients achieved stable disease. | June 5, 2013 | (1)；NCT01869166 |
|  | anti-CD133-CAR vector-transduced T cells | Phase 1/Phase 2 | The patient finally achieved an 8.5-month partial response (PR) from the CART-EGFR therapy and a 4.5-month-lasting PR from the CART133 treatment. | September 4, 2015 | (2)；NCT02541370 |
|  | MUC-1 CART cell immunotherapy | Phase 1/Phase 2 | Clinical trials still in progress | August 16, 2018 | NCT03633773 |
|  | anti-GPC3 CAR-T cells | Early Phase 1 | Clinical trials still in progress | July 6, 2021 | NCT04951141 |
|  |  |  |  |  |  |
| Pancreatic Carcinoma | CART-EGFR | Phase 1/Phase 2 | Of the 14 evaluable patients, 4 achieved partial remission within 2-4 months and 8 had stable disease within 2-4 months. Median progression-free survival was 3 months (range, 4 months) from the first cycle of CAR T-EGFR cell therapy, and median overall survival was 4.9 months (range, 2.9-30 months) in all 14 evaluable patients. | June 5, 2013 | (3)；NCT01869166 |
|  | Anti-hCD70 CAR transduced PBL | Phase 1/Phase 2 | Clinical trials still in progress | July 13, 2016 | NCT02830724 |
|  | CAR-CLD18 T cells | Not Applicable | Clinical trials still in progress | October 5, 2017 | NCT03302403 |
|  | huCART-meso cells | Phase 1 | Clinical trials still in progress | October 27, 2017 | NCT03323944 |
|  | CAR-T cell immunotherapy | Phase 1/Phase 2 | Clinical trials still in progress | August 20, 2018 | NCT03638206 |
|  | CAR modified autologous T cells (CCT301-59) | Phase 1 | Clinical trials still in progress | May 22, 2019 | NCT03960060 |
|  | anti-MSLN CAR-T | - | Clinical trials still in progress | December 18, 2019 | NCT04203459 |
|  | CEA CAR-T cells | Phase 1/Phase 2 | Clinical trials still in progress | April 16, 2020 | NCT04348643 |
|  | CT041(CAR-T targeting claudin18.2) | Phase 1/Phase 2 | Clinical trials still in progress | May 27, 2020 | NCT04404595 |
|  | CT041 autologous CAR T-cell injection | Phase 1/Phase 2 | Clinical trials still in progress | October 9, 2020 | NCT04581473 |
|  | CLDN 18.2 chimeric antigen receptor T cells | Early Phase 1 | Clinical trials still in progress | July 19, 2021 | NCT04966143 |
|  | EPCAM CAR-T | Phase 1 | Clinical trials still in progress | August 31, 2021 | NCT05028933 |
|  | CD276 CAR-T cells | Phase 1/Phase 2 | Clinical trials still in progress | December 3, 2021 | NCT05143151 |
|  | P-MUC1C-ALLO1 CAR-T cells | Phase 1 | Clinical trials still in progress | February 14, 2022 | NCT05239143 |
|  | IM92 CAR-T cells | Early Phase 1 | Clinical trials still in progress | March 11, 2022 | NCT05275062 |
|  | HEC-016 CAR-T cell | Early Phase 1 | Clinical trials still in progress | March 14, 2022 | NCT05277987 |
|  | IM96 CAR-T cells | Early Phase 1 | Clinical trials still in progress | March 18, 2022 | NCT05287165 |
|  | CAR-CLDN18.2 T-Cells (CT048) Autologous Injection | Phase 1 | Clinical trials still in progress | May 27, 2022 | NCT05393986 |
|  | CEA CAR-T cells | Phase 1 | Clinical trials still in progress | May 31, 2022 | NCT05396300 |
|  | CEA CAR-T cells | Phase 1 | Clinical trials still in progress | June 13, 2022 | NCT05415475 |
|  | Claudin 18.2 CAR-T | Phase 1 | Clinical trials still in progress | July 25, 2022 | NCT05472857 |
|  |  |  |  |  |  |
| Gastric Cancer | CAR-T cells targeting EpCAM | Phase 1 | Clinical trials still in progress | June 20, 2018 | NCT03563326 |
|  | CAR-T cell immunotherapy | Phase 1/Phase 2 | Clinical trials still in progress | August 20, 2018 | NCT03638206 |
|  | HER2 specific CAR T cells and CAdVEC | Phase 1 | Clinical trials still in progress | November 14, 2018 | NCT03740256 |
|  | CAR-T/TCR-T cells immunotherapy | Phase 1/Phase 2 | No Results Posted | May 8, 2019 | NCT03941626 |
|  | CAR modified autologous T cells (CCT301-59) | Phase 1 | Clinical trials still in progress | May 22, 2019 | NCT03960060 |
|  | CEA CAR-T cells | Phase 1/Phase 2 | Clinical trials still in progress | April 16, 2020 | NCT04348643 |
|  | CCT303-406 CAR modified autologous T cells (CCT303-406) | Phase 1 | Clinical trials still in progress | August 13, 2020 | NCT04511871 |
|  | chimeric antigen receptor (CAR) T cell therapy | Phase 1 | Clinical trials still in progress | December 2, 2020 | NCT04650451 |
|  | Targeting CD276 CAR T cells | Early Phase 1 | Clinical trials still in progress | April 29, 2021 | NCT04864821 |
|  | EPCAM CAR-T | Phase 1 | Clinical trials still in progress | August 31, 2021 | NCT05028933 |
|  | P-MUC1C-ALLO1 CAR-T cells | Phase 1 | Clinical trials still in progress | February 14, 2022 | NCT05239143 |
|  | Chimeric Antigen Receptor T Cells (CAR-T) targeting claudin18.2 | Phase 1/Phase 2 | Clinical trials still in progress | March 11, 2022 | NCT04404595 |
|  | IM92 CAR-T cells | Early Phase 1 | Clinical trials still in progress | March 11, 2022 | NCT05275062 |
|  | CEA CAR-T cells | Phase 1 | Clinical trials still in progress | May 31, 2022 | NCT05396300 |
|  | Claudin 18.2 CAR-T | Phase 1 | Clinical trials still in progress | July 25, 2022 | NCT05472857 |

**References**

1. Guo Y, Feng K, Liu Y, Wu Z, Dai H, Yang Q, et al. Phase I Study of Chimeric Antigen Receptor-Modified T Cells in Patients with Egfr-Positive Advanced Biliary Tract Cancers. *Clin Cancer Res* (2018) 24(6):1277-86. Epub 2017/11/16. doi: 10.1158/1078-0432.Ccr-17-0432.

2. Feng KC, Guo YL, Liu Y, Dai HR, Wang Y, Lv HY, et al. Cocktail Treatment with Egfr-Specific and Cd133-Specific Chimeric Antigen Receptor-Modified T Cells in a Patient with Advanced Cholangiocarcinoma. *J Hematol Oncol* (2017) 10(1):4. Epub 2017/01/07. doi: 10.1186/s13045-016-0378-7.

3. Liu Y, Guo Y, Wu Z, Feng K, Tong C, Wang Y, et al. Anti-Egfr Chimeric Antigen Receptor-Modified T Cells in Metastatic Pancreatic Carcinoma: A Phase I Clinical Trial. *Cytotherapy* (2020) 22(10):573-80. Epub 2020/06/13. doi: 10.1016/j.jcyt.2020.04.088.
